# Why we have duties of autonomy towards marginal agents

**DOI:** 10.1007/s11017-023-09623-5

**Published:** 2023-05-12

**Authors:** Anna Hirsch

**Affiliations:** Institute of Ethics, History and Theory of Medicine, Ludwig-Maximilians-Universität München, Lessingstr. 2, D-80336 München, Germany

**Keywords:** Autonomy, Autonomy rights, Value of autonomy, Marginal agents, Caring attitudes

## Abstract

Patients are usually granted autonomy rights, including the right to consent to or refuse treatment. These rights are commonly attributed to patients if they fulfil certain conditions. For example, a patient must sufficiently understand the information given to them before making a treatment decision. On the one hand, there is a large group of patients who meet these conditions. On the other hand, there is a group that clearly does not meet these conditions, including comatose patients or patients in the late stages of Alzheimer’s disease. Then there is a group of patients who fall into the range in between. At the lower end of this range are so-called ‘marginal agents,’ which include young children and patients in the middle stages of Alzheimer’s disease. They also do not meet the typical requirements for autonomy, which is why they are usually granted fewer autonomy rights. However, some of them are capable of ‘pre-forms’ of autonomy that express what is important to them. These pre-forms differ from mere desires and reflect the identification/authenticity condition of autonomy. They have something in common with autonomous attitudes, choices, and actions – namely, they express the *value* of autonomy. As I will argue, autonomy is a value worthy of protection and promotion – even in its non-reflexive forms. Against this background, it becomes clear *why* we have autonomy duties, more precisely positive, autonomy-enabling duties, towards marginal agents and why we should give them as much attention as autonomy duties towards competent patients.

## Introduction

I would like to begin by illustrating two case-scenarios that will be referenced throughout the paper:

46-year-old Helen was diagnosed with breast cancer two years ago. After several chemotherapies, which she experienced as very exhausting and did not lead to the hoped-for success, she decides against another chemotherapy. However, her physician advises her to continue chemotherapy and makes it clear to her that foregoing therapy will significantly reduce her chances of being cured. Helen, however, sticks to her refusal, about which she has also consulted with her husband and her psychotherapist. She does not want to spend more time in hospitals and continue suffering from the side effects of the chemotherapy.

75-year-old Martha has been suffering from Alzheimer’s disease for five years.^1^ She is no longer able to name the day of the week, the month, or the year. It is also becoming increasingly difficult for her to find her way around the day care centre she has been attending for two years. Despite all this, she regularly takes part in research projects in the context of Alzheimer’s research. When asked about her participation, she says things like, “Of course I could have refused, believe me, but if I can help myself and others, I will.”^2^ After participating in research projects, Martha always seems particularly satisfied. In contrast, she reacts aggressively when she is denied participation. However, in view of Martha’s state of health, which is very critical on some days, the healthcare providers (HCPs) wonder whether they should continue to allow her to participate.

In saying that patient autonomy must be respected, and that autonomy is a thick normative concept,^3^ one usually thinks of cases such as Helen’s: a competent and informed patient refuses a treatment recommended by a physician. Since Helen is autonomous, she has the right to refuse the treatment and her physician has the duty to respect her decision. Autonomy in this context serves as a negative right of defence.^4^ This ‘normative function’ of autonomy is often brought to the fore in medical ethics literature. In the following, however, the focus is on the second case study and the normative meaning of autonomy that becomes apparent through it. I am particularly interested in so-called “marginal agents” (i.e., patients like Martha), who find themselves ‘at the margins’ of autonomy and whose autonomy is doubted.^5^

Both in medical ethics and in medicine itself there is broad consensus that one should consider the interests, preferences and wishes of only rudimentary or non-autonomous patients in therapy decisions.^6^ Reasons given include the well-being of the patients [11, 12], the moral value of preferences [13, 14] or – especially in the case of children – promoting the development of autonomy [15, 16]. Despite this consensus, empirical literature shows that there is still a demand for improving the participation of marginal agents in patient care (e.g., [17–22]).^7^

In addition, marginal agents are not always given sufficient attention in medical-ethical books on patient autonomy (e.g., [23, 24]). This also applies to the ‘standard work’ of medical ethics, *Principles of Biomedical Ethics*, which addresses respect for autonomy as a central principle of medical ethics [25]. That in such a renowned work (widely used not only in academic medical ethics but also in clinical ethics, and already in its eighth edition in 2019), marginal agents are addressed almost exclusively in the context of proxy decision-making may give the false impression that apart from this, one has no duties of autonomy towards them. The fact that the two authors, Tom L. Beauchamp and James F. Childress, also comment in precisely this direction is something I will address later.

One reason why the two authors do not address the margins of autonomy in more detail may be that they focus on the normative meaning of autonomy as a right. Marginal agents are not usually granted autonomy rights, such as the right to informed consent. However, autonomy rights are based on the assumption that autonomy is something valuable and thus worth protecting in individuals’ lives. And, as I will argue, this also applies to only rudimentarily or not (any longer) autonomous persons. One can thus place autonomy duties towards marginal agents on the same normative basis as rights one grants to autonomous persons. Another reason for the omission of Beauchamp and Childress may be that they commit themselves to an understanding of autonomy that presupposes rational capacities [17, p. 369]. These are usually not met by marginal agents. However, there are other accounts of autonomy that place more emphasis on non-rational qualities such as emotions.

And it is precisely on these two aspects, the value of autonomy and the importance of non-rational forms of autonomy, that I would like to draw on to strengthen the position that we have duties of autonomy^8^ towards marginal agents. These duties are different from the negative, but also from the positive autonomy duties one has towards clearly autonomous patients like Helen, yet they can demand much more. As already indicated, I will argue that these duties are based on the value of autonomy – only referring to well-being or the moral value of preferences is not sufficient in my view.

To substantiate my theses, it is first necessary to emphasise more clearly what distinguishes marginal agents from non-marginal agents (i.e., competent and clearly autonomous agents). This is of course related to the question of what kind of autonomy marginal agents are still or are already capable of. Following Agnieszka Jaworska, I will argue that marginal agents are capable of a non-reflexive form of autonomy, so-called “carings” or “caring attitudes.” I will show what distinguishes these attitudes from mere desires and interests and argue that they give rise to duties of autonomy towards marginal agents. The fact that we also have duties of autonomy towards these patients can be underpinned by the value of autonomy, which I will then discuss in more detail. Lastly, I will present examples of autonomy-enabling duties towards marginal agents and discuss a selection of possible objections to the ideas presented.

## Marginal and non-marginal agents

One could say that Helen is a ‘prime example’ of an autonomous agent; she is not only fully informed by her physician and gives reasons why she refuses therapy, but she has also reflected on her decision together with others. She would probably be considered autonomous not only according to the so-called ‘standard view of autonomy’ in medical ethics (the *three-condition theory* of intentionality, understanding and voluntariness which Beauchamp and Childress develop in *Principles of Biomedical Ethics* [25, p. 102]), but also according to most of the more sophisticated theories of autonomy discussed in the philosophical debate.

However, I need to say a little more about the autonomy of non-marginal, competent agents like Helen to be able to distinguish her from marginal agents like Martha. First of all, I am chiefly concerned with autonomy exercised by individuals in their decisions, actions, and in the way they lead their lives. By autonomy, I mean what is often described as individual autonomy or self-determination rather than a moral understanding of autonomy (for example in the sense of Immanuel Kant).^9^ Here I mainly focus on local autonomy – broadly speaking, the ability to decide and act autonomously.^10^ In order to be considered autonomous in this sense, agents must fulfil a number of conditions, which are defined differently by different theories of autonomy.^11^ In the following, I consider the following abilities, which are shared by many theories, as central to autonomy.

Firstly, autonomy presupposes a certain degree of *rationality and understanding*: Helen understands the situation she is in, the possible courses of action, and their consequences. Secondly, autonomy is a matter of being in control of one’s own actions and decisions [32, pp. 12–13]. Helen’s decision must be sufficiently *voluntary*. Of course, people are subject to numerous external influences that affect their decisions and actions. However, these must not be so strong that a person feels externally controlled and is no longer able to reflect on these influences. The same applies to internal influences, like an obsessive-compulsive disorder. Helen is neither driven to her decision by her husband nor by internal psychological constrains. Thirdly, one must be able to *actively exercise* one’s own autonomy [33, p. 712]. Helen expresses that she no longer wants to have chemotherapy. She is not only able to decide that for herself, but also to communicate it. The fourth and final condition is more demanding: an autonomous decision or action must also be carried out ‘for one’s own’ reasons or values. This condition exceeds the voluntariness condition because it is not only about not being controlled, but about being ‘true to oneself’ and acting according *to what one identifies with*, what is important to oneself. In this context, there is often talk of the ‘identification’ or ‘authenticity condition’ of autonomy [32, pp. 13–14], which is defined differently by different theories of autonomy. According to Harry G. Frankfurt’s hierarchical account, for example, a desire is authentic when it has been reflected and accepted at a higher level of reflection [34]. Without further context, it is difficult to judge whether Helen also meets the identification condition. However, it can be assumed that she fulfils it to a sufficient degree. This leads me to another important assumption that I will rely on: like most authors in the debate, I understand autonomy to be a matter of degrees [25, p. 103; 35, p. 391; 36, p. 7; 37, pp. 415–416]. That means, in general terms, that persons, actions, and decisions can be more or less autonomous. Without question, much more could be said about the concept and conditions of autonomy. However, this brief outline is sufficient for the following considerations.

In order for autonomy to unfold the normative meaning mentioned at the beginning of the paper, it must be determined to which degree of realisation this normative meaning applies. ‘To what extent’ must an action or decision be autonomous in order to be respected? ‘To what degree’ must a person realise her capacity for autonomy in order to be granted autonomy rights? It is not easy to give clear answers to these questions. Beauchamp and Childress, for example, avoid the determination of thresholds by stating that they can only be determined in considering the concrete decision-making context [25, p. 103]. I will not give a clear answer here either. For my purposes, it is sufficient to assume that one can usually expect patients to be above the crucial threshold to count as autonomous. Nevertheless, there are some groups of patients who are clearly below this threshold, including Alzheimer’s patients in the late stages of the disease and comatose patients. They are usually granted fewer autonomy rights than competent patients.^12^

However, as mentioned above, another group of patients falls into the range between ‘sufficiently autonomous’ and ‘clearly not autonomous.’ At one end of this spectrum are marginal agents. In addition to Alzheimer’s patients in an intermediate stage of the disease, such as Martha, young children^13^ and sometimes patients with mental illnesses – such as addictive disorders – fall into this category. What now makes me suspect that Martha may be within this range? Even though she suffers from significant cognitive impairment and would fail the autonomy conditions mentioned earlier, she displays something that is not visible in the behaviour of late-stage Alzheimer’s patients as well as comatose patients: *a deep concern for something*, namely for participation in research. It is not a short-term, mundane kind of caring about something, like caring about getting a coffee, but a long-term kind that expresses what is important to her and thus reflects the identification/authenticity condition of autonomy. It is with this kind of caring that I deal with in the following section.

## Caring as manifestation of autonomy

According to most theories of autonomy, identification with a desire or an attitude in general presupposes a reflexive process, such as the reflection of the attitude by higher-order desires [28, 34], long-term self-governing policies [40], one’s valuational system [41], or against the historical process that gave rise to the attitude [42]. According to Jaworska, this view of identification contradicts the fact that marginal agents who do not have the required reflective skills identify with their carings^14^ – not through reflection, but through complex, emotional attitudes. Martha, for example, reacts aggressively when she is denied participation in research projects and happily when she is allowed to participate. What is meant by caring as a complex, emotional attitude is made clearer by the following example:

Three-year-old Paul got hamster Fluffy from his parents a few weeks ago. Even though Fluffy is nocturnal, he sometimes shows himself during the day, which brings Paul great joy, so that he immediately calls his parents and spends hours at Fluffy’s cage. When Fluffy does not show himself and hides in his little house, Paul is sad and asks his mother if Fluffy is perhaps ill. As Paul goes on holiday with his parents and his grandmother is supposed to look after Fluffy, Paul makes her swear to look at his cage every day to see if Fluffy is still there.^15^

According to Jaworska, carings are composed of various, more or less complex, emotional inclinations and desires towards the object of caring [43, pp. 89, 100; 7, pp. 559–560]. Feelings, desires, and plans arise from the importance attached to the object. They are not feelings that come over one suddenly, like anger or fear, and from which one can distance oneself. One feels more connected to carings than to only temporary emotions: “Because they connect various aspects of our psychology together, and support our psychological unity and continuity over time, carings are tied to our sense of self more closely than other attitudes – they are more strongly our own” [43, p. 92]. This corresponds to the attitude Paul has towards his hamster; unlike his desire for sweets, which Paul occasionally expresses in the morning, he does not forget Fluffy after one hour. He shows a series of emotional reactions directed towards one object, the hamster, over a longer period of time. Furthermore, he can recognise the importance of Fluffy and derive stable intentions and plans from it, such as giving instructions to his grandmother.

Following Jaworska, agents like Martha and Paul can relate to an object in a way that expresses more than just a temporary desire. Caring is a deeply rooted and complex emotional attitude towards an object that expresses what is important to an agent. And assuming that Paul and Martha feel more connected to their carings than to other desires they have, experiencing a disappointment of these carings is probably worse than experiencing a disappointment of any of their desires. That is, when Paul’s grandmother refuses to swear to him, she violates what is important to him to a greater extent than when she denies him sweets in the morning. Since carings are not just any desires, but longer-term attitudes from which agents derive a sense of self and motivation(s) for action, they can be considered a manifestation of autonomy [43, pp. 93–95; 37, pp. 415–416], which reflects the identification/authenticity condition of autonomy.^16^ Of course, Paul is not capable of reflexively relating to the object of his carings, for example, of judging whether his worry that Fluffy might simply disappear is well-founded. But as shown, this is not necessary since identification can also occur through emotions.

One might say – okay, fair enough, Paul and Martha may show first (or last) signs of autonomy, but neither of them can be considered truly autonomous since they fail the aforementioned conditions of autonomy as well as the conditions of most theories of autonomy. Therefore, one can only speak of marginal agents as having interests – some of which are longer-term and more important to them than others – so there is no need to introduce the concept of autonomy in this context.^17^ In response, there are at least four reasons for referring to caring attitudes not as mere interests, but as sincere manifestations of autonomy:

1. Simply describing carings as ‘interests’ would not do justice to the special identity-forming function of carings, which distinguishes them from mere desires and mundane appetites.
2. In addition, it could lead to confusion with objective well-being interests; for example, the interest in not having to endure pain, which are usually taken into account by the best interests standard (BIS).^18^
3. Understanding carings as manifestations of autonomy makes it explicit that these attitudes are worthy of respect and promotion – not with a view to the well-being, but the (developing/still existing) autonomy of marginal agents.
4. This in turn suggests that it is not justified to pass over carings without good reason and to simply treat marginal agents in a paternalistic way. Paternalism is usually opposed to autonomy – and thus also opposed to carings as particular manifestation of autonomy – and must therefore be carefully considered.

These points suggest that we have not only duties of nonmaleficence and beneficence towards marginal agents, but also duties that are to be regarded as duties of autonomy. The carings of Martha and Paul have something in common with Helen’s clearly autonomous decision – as a manifestation of autonomy, they express the value of autonomy, which is to be protected and promoted. But before elaborating on what kind of autonomy obligations we have towards marginal agents, I would first like to look more closely at the value of autonomy as it clearly affirms that we have autonomy obligations towards marginal agents.

## The value of autonomy

If something is valuable, there are reasons to protect and promote it – in one’s own life, but also in the lives of others. Autonomy is a central value in modern societies. The fact that democracies exist, that children are educated to be independent agents, and that self-fulfilment is considered an ideal are all indications of the acceptance of the value of autonomy. Therefore, the interesting question is not whether autonomy is a value, but what exactly makes autonomy valuable. There are many different reasons *why* people may consider autonomy a value. One reason is the esteem and recognition one receives from others as an autonomous person, which expresses the instrumental value of autonomy – autonomy is valued *as a means to* other valuable things, such as self-fulfilment, recognition and self-development [52, p. 130]. Most often, however, the instrumental value of autonomy is discussed in terms of well-being or the good life. Sometimes the relationship is understood as autonomy being *a means to* a good life or to well-being – which is different from the view that autonomy forms a central component of well-being, for example as part of an objective-list theory of well-being [53, p. 254; 54].

If one ascribes autonomy only as an *instrumental value* in terms of its contribution to other things, such as well-being, then it is questionable whether one can ascribe this value to the caring attitudes of marginal agents. Marginal agents often do not know what contributes to their overall well-being.^19^ Martha, for example, cannot decide for herself on which days her condition allows her to participate in Alzheimer’s research and on which days participation is contrary to her well-being. If one considers autonomy *solely* valuable as a means to realise well-being (or a good life),^20^ one could consequently hardly justify that autonomous decisions and actions that are contrary to one’s well-being are also valuable [55, pp. 266–267; 56, pp. 50–51]. But would one not also want to say that sometimes one is simply concerned with deciding and acting for oneself, even though one is unsure whether this really contributes to one’s overall well-being? In such situations, one seems to prioritise the value of autonomy over other values and goods (e.g., health). Therefore, the value of autonomy cannot be exhausted in its contribution to other values, such as one’s overall well-being. It seems, instead, that a value is ascribed to autonomy *for its own sake*. I will argue for this view in the following paragraphs.

That one values deciding, acting, and living autonomously as such (independently of its consequences and merits) is already displayed by small children – as soon as they are able to do something themselves, they absolutely want to do it themselves [57, pp. 101–102]. Steven Wall tries to back this up with a thought experiment: imagine that one could have a well-meaning advisor on one’s side who knows all their talents, desires, etc., and always wants the best for them. Would they want to put their lives in the advisor’s hands? According to Wall, most would refuse this offer. Even if one hands over responsibility in particular areas of one’s life to experts, it is part of a good human life that one leads it themselves and gives personal meaning to it [52, pp. 146–147].^21^ So, it again seems that autonomy is valued for its own sake, as a *final value*.^22^

Valuing autonomy for its own sake does not only mean valuing it regardless of consequences and the realisation of other values, but also regardless of whether the person can actively exercise her autonomy or is – largely or completely – dependent on the help of others to do so. If autonomy is valued for its own sake, then it is already valuable in its beginnings and preliminary forms; it is valuable as soon as qualities are present that speak for autonomy (such as the identity-forming function in the case of carings). And it follows that autonomy should also be protected and promoted in these forms. Therefore, the final value of autonomy seems particularly important for establishing duties of autonomy towards marginal agents. Now, of course, there is no point in granting marginal agents like Martha autonomy rights such as the right to informed consent (IC).^23^ She does not have the necessary capacities to adequately understand and assess information. Consequently, granting her this right would not express any appreciation of her remaining autonomy. The focus must therefore be on *promoting* the value of autonomy expressed in her carings. In this case, the normative meaning of autonomy is not to be understood in terms of a negative defensive right, but as a positive right; a right to be enabled to exercise (still existing/developing) autonomy.

That is, if a patient is already showing ‘signs’ of autonomy or is still capable of expressing specific forms of autonomy such as caring attitudes, then one has positive, autonomy-enabling duties towards the patient that are rooted in the value of autonomy – even if it cannot be said that the patient fulfils the conditions for autonomy and is accordingly granted autonomy rights like the right to IC. Of course, one also has positive duties of autonomy towards non-marginal, competent agents like Helen. For example, a patient who is generally capable of autonomy can only exercise her right to IC if she has been fully informed. The positive duty then consists of enabling the patient to make an autonomous treatment decision through careful and patient-oriented disclosure. However, this cannot be what is meant by the positive duties we have towards *marginal* agents. I would therefore like to address these duties in more detail below.

## Autonomy-enabling duties towards marginal agents

In its conference report *Autonomy and relationship* (2016), the *Swiss Academy of Medical Sciences* (SAMS) writes:

Children and people with disabilities or dementia also have a right to be heard in medical decision-making. It makes a crucial difference to their perception of self-efficacy whether they have their say or not. This is where the health professionals’ duties of empowerment in the context of relational autonomy come into play: respect for the patient’s autonomy includes promoting and supporting the person concerned in his or her capacity for autonomy [66, p. 58].^24^

In this context, the SAMS speaks also of “Befähigungspflichten” (enabling duties), which are strongly rooted in a relational understanding of autonomy [66, pp. 11, 23]. I agree with SAMS here in both respects; namely, *that* these enabling duties *exist* (I have so far referred to them as ‘autonomy-enabling duties’) and that they emphasise the importance of a *relational understanding* of autonomy in medical ethics. Autonomy empowerment happens through others, through their active intervention(s), through relationships. A relational understanding of autonomy, as advocated prominently by feminist philosophers (see for example [62, 66–69]), allows one to see relationships as a constitutive component of the exercise of autonomy.^25^ External interventions need not threaten autonomy; rather, they can make it possible in the first place. I think the cases discussed so far are good examples of this. However, they also show that autonomy-enabling duties can demand very different things from someone.

While Martha is dependent on support especially regarding the articulation and active implementation of her carings, Paul requires encouragement in his attempt to exercise autonomy and offering him opportunities to develop it further. One probably has the ‘smallest effort’ with Helen. As already mentioned, she needs to be sufficiently informed. From a relational autonomy perspective, it is also conducive to her autonomy that she discusses the decision with her husband and her psychotherapist – so long as they reflect together with her and do not put any undue pressure on her. Providing information and joint reflection are positive duties of autonomy, which can be referred to as “decision-making assistance” [66, 70].

### Decision-making assistance

Regarding patients with severe cognitive impairments, there can be no talk of decision-making assistance in the sense of providing information and having a joint conversation. With a view towards promoting and respecting the value of autonomy, it would be important to further develop methods of how they too can be enabled to take part in the decision-making process. How this can be achieved is not only a question of how the information is conveyed (e.g., in particularly simple terms), but also *in what setting* and *by whom*. One possibility is, of course, to support the decision-making of Alzheimer’s patients – but also of children – with illustrations or other visual aids.^26^ This is already practised, for example, at the University Hospital in Geneva within the framework of the *Handicap HUG 3* programme (here with the target group of adult patients who have an intellectual disability, autism spectrum disorders or multiple disabilities).^27^ This programme encompasses, among other things, greater involvement of caregivers and illustrative educational methods, such as comics explaining basic medical terms and procedures [72, pp. 28–29].

Equally relevant, however, appears to be the relationship with health care providers (HCPs) and the time invested in providing information. Involving patients like Martha in the decision-making process – to find out, for example, whether she really wants to take part in a certain research project – can require a considerable amount of time. Enough time, illustrations, extra trained staff, and a trusting relationship with the HCPs are certainly helpful tools to involve marginal agents in decisions. Decision-making assistance that does justice to the value of autonomy refers to a patient’s specific situation, her abilities, and individual competences. In addition, the SAMS together with the Swiss *National Advisory Commission on Biomedical Ethics* (NCE) rightly points out that practical things can also support autonomous decision-making, such as interpreters and visual or hearing aids [70, p. 8]. This shows that it is also important that the value of autonomy is anchored even more strongly in the institutional structures of the healthcare sector.

### Supporting the realisation of carings

In Martha’s case, however, even a comprehensive form of decision-making assistance is not enough. She is also dependent on support in the implementation of her carings. As shown above, being autonomous means also *exercising* one’s autonomy (i.e., not only forming autonomous desires and attitudes, but also articulating them and putting them into practice). Martha can express her caring to participate in Alzheimer’s research through emotional reactions and sometimes also through statements, but she can neither register for research projects nor appear in the right place at the right time. In the case of marginal agents like Martha, fulfilling positive autonomy duties therefore requires a much higher level of commitment. It may involve taking on the active implementation of their carings almost entirely. The additional resources that must be spent on this are justified because, as shown, autonomy is a final value worthy of protection and promotion, even in its non-reflexive forms. After all, are great efforts not also made for the preservation and promotion of well-being, another central value in patient care? Why then should it not be equally justified to make comparable efforts with a view to upholding the value of autonomy?

Paul, on the other hand, is to a certain extent already capable of articulating and actively implementing his carings: he asks his parents if Fluffy is sick, he urges his grandmother to look after Fluffy, etc. Unlike Martha, Paul is not progressively losing his autonomy skills, but is in the process of gradually developing them. However, in order to become an autonomous agent and live an autonomous life, Paul is dependent on others to foster these skills. Thus, the promotion of skills necessary for autonomy is another positive autonomy obligation, which becomes more extensive with marginal agents, especially children.

### Promoting children’s autonomy

The promotion of children’s autonomy is a central topic in medical ethics as well as in philosophy in general, to which I cannot do justice here.^28^ However, even in the context of this topic, it is often overlooked that it is the *value* of autonomy that justifies positive duties of empowering children’s autonomy. Especially in the medical context, where children’s voices are sometimes still too little heard [20, 22, 74–77], the value of autonomy once again makes it quite clear that greater efforts in this regard are warranted. In the case of therapy decisions that affect the children themselves, there is of course always the added difficulty that one has a greater responsibility towards children in terms of their well-being [78, p. 184]. However, the final value of autonomy speaks in favour of also paying attention to children’s autonomy – also in the form of carings – within the context of their therapy.

Now, of course, Paul’s case is not about a therapy decision. Nevertheless, I would like to return to it briefly to clarify the question of what autonomy obligations one has towards him. The main question is how one should react to Paul’s carings and whether one can possibly take them as an opportunity to further strengthen his autonomy capacities. As has been shown, carings are a valuable manifestation of autonomy. And it is a widespread and uncontroversial view in family ethics that it is the duty of parents to enhance the autonomy of their children [79, 80, 55, 37, p. 49]. Both Paul’s parents and his grandmother would not fulfil this duty if they did not take Paul’s carings seriously and dismissed them as exaggerated or even made fun of them. Hereby, they would not pay attention to Paul’s carings and therefore Paul would probably feel that his concerns are not worthy of being taken seriously, even though he has drawn the right conclusion; if something is important to me, I act in its favour. In contrast, a reaction that expresses appreciation for Paul’s carings that also promotes his autonomy skills would be to give him recognition for his actions. In concrete terms, not ridiculing his concern that Fluffy might disappear, but assuring him to check on Fluffy and his well-being every day. Of course, this does not preclude explaining to Paul that Fluffy cannot disappear from the cage. Nevertheless, it must be signalled to him that his concern for his hamster and the consequences he draws from it are justified.

Paul’s efforts for the well-being of his hamster could also be described as a kind of ‘project’ that is important to him and that he has been pursuing for a while now. According to Monika Betzler, projects – and prototypes of projects^29^ – foster the ability to value in children and thus create an important prerequisite for the authenticity condition of autonomy. She writes: “caring […] is the basis of valuing because through it children guide their receptivity to the value of an object or state of affairs, in relation to their perspective, and in a more stable fashion. They thereby experience themselves as being susceptible and vulnerable to that object or state of affairs” [55, p. 75]. Within the framework of projects, children could devote themselves to tasks that are important to them for a longer period of time. They learn what it is like to commit to something and what feelings can be associated with it (e.g., joy when their project succeeds, frustration when things do not go so well). In the longer term, stable personal values can develop from these project-experiences to which children can also reflexively relate as they grow older.

Now, what can be derived from this regarding the medical context? If one considers children with chronic illnesses that require them to spend a lot of time in hospital from birth (e.g., congenital heart defects or mucoviscidosis), it can make sense to involve them in (proto-) projects as part of their treatment as well. This seems especially important if their illness hardly gives them this opportunity outside the hospital; if they cannot, for example, look after a pet, join a football club, or build igloos in the snow. The final value of autonomy indicates that one already has an obligation towards young children to give them the opportunity to form carings and pursue (proto-) projects. Children, who are already disadvantaged by their illness, must not be denied this opportunity. How this would look in practice remains to be worked out. As in the case of Alzheimer’s patients, the promotion of children’s autonomy in the context of their healthcare – for any case – presupposes external conditions that support autonomy, such as specially trained HCPs.

Comprehensive decision-making assistance, supporting the implementation of caring attitudes, and promoting autonomy skills are unquestionably only examples of positive autonomy obligations towards marginal agents. They also need to be further elaborated for their implementation in medical practice. Nevertheless, it has become clear why we have positive duties of autonomy towards marginal agents and that they can demand more of us than positive duties of autonomy towards non-marginal agents. As I have argued, these duties can be justified by the value of autonomy. However, in order to reinforce these claims, I would like to anticipate at least three possible objections.

## Objections and replies

### The superfluity of referring to autonomy as a value

One might argue that the requirement to support marginal agents in exercising their autonomy is already captured by the understanding of autonomy as a positive right. Therefore, the reference to autonomy as a value seems superfluous. Even Beauchamp and Childress, who focus on the decision-making rights of competent patients, point out that autonomy not only carries a negative duty not to interfere unjustifiably in the actions and decisions of others, but also a positive duty to support others in their exercise of autonomy [25, p. 104]. According to them, however, one only has these obligations towards patients who are competent to act and decide autonomously. They write: “Obligations to respect autonomy do not extend to persons who cannot act in a sufficiently autonomous manner and to those who cannot be rendered autonomous because they are immature, incapacitated, ignorant, coerced, exploited, or the like” [25, p. 105]. As examples, the two authors cite young children, irrationally acting suicidal patients, and drug addicts [25, pp. 105–106]. In their discussion of the principle of respect for autonomy, they focus on the one hand on the obligation towards clearly competent and autonomous patients [25, pp. 104–112] and on the other hand on patients who are clearly no longer autonomous. With regard to the latter, they understand respect for autonomy mainly as respect for *precedent autonomy* – i.e., for preferences expressed earlier in a competent state [25, pp. 139–143].^30^ What it means to respect the autonomy of patients with diminished autonomy, by contrast, they do not address. This leads, among other things, to neglecting patients with mild or moderate dementia, as Hojjat Soofi has pointed out [84].

This omission is, in my opinion, based on a rationalist understanding of autonomy and on the concept of autonomy as a right. The problem with focusing exclusively on the normative meaning of autonomy as a right – even considering its meaning as a positive right – is that the attribution of autonomy rights is usually linked to the fulfilment of certain conditions.^31^ In order for a person to be granted these rights, she must fulfil the necessary conditions (understanding, voluntariness, etc.) to a sufficient degree. Marginal agents do not meet these conditions, they “cannot act in a sufficiently autonomous manner.” But, as I have shown, they are capable of non-reflexive forms of autonomy that should not simply be dismissed as mere volatile desires. Caring attitudes have something in common with the autonomous attitudes and desires of competent agents like Helen; they display the value of autonomy, which is considered worthy of protection and promotion – independent of the degree of realisation of autonomy. In order to justify positive autonomy obligations towards marginal agents, it is therefore not superfluous but *necessary* to refer to the value of autonomy as manifest through carings.

### The problem of defining boundaries

A subsequent objection could be that the justification with autonomy becomes inflationary if every form or manifestation of autonomy, however rudimentary it may be, is evaluated as an expression of the value of autonomy. Thus, concrete thresholds would have to be defined as to when the attitude of a marginal agent qualifies as a manifestation of autonomy and establishes positive autonomy obligations. Although I have shown what distinguishes caring attitudes from everyday desires and mere appetites, the question arises whether these can always be clearly distinguished from each other. For example, how stable does an attitude have to be in order to be considered as a caring attitude? Is it enough if Paul cares for Fluffy for a day or does it have to be at least a week? As one expands the scope of autonomy-enabling duties, such questions will certainly arise, and they are undoubtedly justified.

With regard to setting thresholds for autonomy, I would first like to emphasise that this is a general problem in dealing with patient autonomy. Thus, the objection of unclear thresholds could be raised against almost all theories that conceptualize autonomy as a matter of degree. Some authors try to circumvent this problem by pointing out that thresholds cannot be set in general terms, but only in the specific context. For example, they argue that the degree to which a decision or action and its consequences must be understood depends on its complexity [25, pp. 102–103, 117, 131] or the risks it poses to the well-being of an agent [47, p. 55; 85, p. 139]. However, these proposed solutions come with their own problems [27, p. 192]. How complex a decision is, for example, hardly seems to be generally ascertainable from an external perspective but is dependent on the subjective experience of the patient.

However, I do not understand the setting of thresholds as a major problem in my argument since I have argued that autonomy is valuable for its own sake regardless of its contribution to other values. As this refers also to *rudimentary* forms of autonomy, one has the duty to protect and promote this value as soon as one notices signs of autonomy in another person’s actions and decisions – even if it means acting against well-being considerations or the future autonomy of a person. Of course, a trade-off must always be made, as autonomy is not an absolute value, and in the best case, actions should be taken in a way that protects *both* well-being *and* autonomy interests. I explicitly oppose the position that the preceding autonomy of a patient (e.g., in form of a living will) [25, pp. 139–143] or their future autonomy (e.g., in the case of children) has greater value. Joel Feinberg, for example, argues that the restriction of the current will of children is usually justified if it is a matter of protecting the autonomy of the adults the children will become [86, p. 78]. Although this may be the case – how often depends on the age of the child, the situation, and much more – the value of children’s autonomy as children [74, p. 18] should always be regarded. Nevertheless, I think most would agree that non-reflexive and non-rationalistic forms of autonomy such as carings already reflect the value of autonomy and thus give rise to autonomy-enabling duties.

More difficult, however, is determining what kind of autonomy-enabling duties apply to which groups of patients and, of course, how these duties should be balanced against other duties, such as beneficence. As the case studies have shown, autonomy-enabling duties demand very different things in different cases. Further differentiating these duties regarding certain patient groups and certain situations is undoubtedly an important task, but one that I cannot pursue further here.

### Objection of overburdening

The examples I have given of autonomy-enabling duties already suggest that their implementation can be challenging. A legitimate objection could therefore be that the implementation of autonomy-enabling duties towards marginal agents would require an unrealistic amount of additional time and personnel and financial effort. This would be a problem that concerns institutional framework conditions in the healthcare sector. In addition, it could be pointed out that it is a professional overburden for HCPs. After all, it is usually assumed that the main task of HCPs is to protect and promote the health of patients.^32^ Of course, they are also obliged to respect patients’ rights, such as the right to IC. But is it not too much to ask that they do everything possible to promote autonomy as a value? It would certainly be easiest to treat Martha according to objective well-being considerations and decide for her which research projects she should be allowed to participate in.

In my opinion, the fundamental question here is what importance one gives to autonomy in patient care. Because even if it would be unquestionably challenging to orient medical action more strongly towards the value of autonomy for marginal agents, it would not be impossible. Projects like the *Handicap HUG 3* programme at the University Hospital in Geneva demonstrate that even marginal agents can be more involved in treatment decisions. If one concludes that the value of autonomy must be given more space in patient care, then it would also be justified to devote more resources to promoting this value. The focus would then be on establishing autonomy-promoting structures in the healthcare sector [70, p. 12]. This would include, for example, training HCPs specifically in conversation skills aimed at supporting autonomy and strengthening participatory decision-making. It would also mean scheduling more time for doctor-patient conversations and developing methods to best elicit and respect the interests of marginal agents – just to name a few examples. Of course, this would require a lot of work and addressing complex challenges – but it seems to be within the realm of possibility to strengthen autonomy as a value in patient care. How many resources should be put into this project is a question that would need to be discussed in more detail.^33^ Therefore, in view of the value of autonomy, I think it is at least clear *that* it would be justified to put more effort into it.

Now, however, the expansion of autonomy-enabling duties could also be seen as overburdening the marginal agents themselves. The concern expressed, amongst others, by Marina Oshana that one might value autonomy “too much” and, on that basis, force non-autonomous persons to be more self-directed and overburden them [29, pp. 100, 103] is to be taken seriously. But since there is also the reverse danger of treating marginal agents paternalistically by not listening to their concerns and deciding from one’s own point of view what is best for them, it is probably a question of looking carefully at each individual case and finding the right middle ground. In this case, the task is to weigh patient autonomy (or carings) against other well-being interests of patients, such as health. Regarding autonomy-enabling duties, I would like to stress that they are not *absolute* duties, but *prima facie* duties, which must therefore always be carefully weighed against other duties.

While I admit that all three objections have some merit and undoubtedly need further discussion, I hope to have once again reinforced my point of view that autonomy as a value should have greater importance in dealing with marginal agents and patients in general.

## Conclusion

In this paper I elaborated on autonomy-enabling duties towards marginal agents like Martha and Paul. I argued that the normative meaning of autonomy as a value is to be protected and promoted in this context. The attribution of autonomy rights to patients, such as the right to IC, is usually tied to certain preconditions that marginal agents do not fulfil. Nevertheless, marginal agents express attitudes – so called caring attitudes – that should not be dismissed as mere desires and mundane appetites, but should be understood as windows that show what is really important to them. Since carings represent complex, emotional attitudes with an identity-forming function, they are a manifestation of autonomy worth respecting. Further, I argued that autonomy is valuable for its own sake – regardless of its consequences and of its degree of realisation. Caring attitudes can therefore also be considered as an expression of the final value of autonomy. And if one wants to protect and promote the value of autonomy, one should consequently promote caring attitudes, either through supporting marginal agents in articulating and implementing their carings or in the development of autonomy skills. I have indicated what this can demand from HCPs and those who care for marginal agent patients. However, there is still a lot to do, especially in terms of the formulation, implementation, and weighing of concrete autonomy-enabling duties towards marginal agents vis-à-vis other duties in the healthcare context, such as duties of beneficence. Regarding this, a collaboration between philosophers, medical ethicists, and medical practitioners would, in my opinion, be a promising project.

## Footnotes

1 Alzheimer’s disease (AD) is the most common form of dementia. Dementia leads to a decrease in cognitive abilities [1, pp. 299–300]. AD is usually differentiated into three stages [1, p. 300]. Martha is currently assigned to the second stage, moderate AD: “Independent living is only possible with considerable restrictions and with the support of others” [1, my own translation].

2 The example is based on a case that Agniezska Jaworska discusses in her paper *Respecting the margins of agency: Alzheimer’s patients and the capacity to value* [2, pp. 117–118], which was originally taken from a study by Steven R. Sabat [3, p. 46].

3 By “thick normative concepts” or “thick moral concepts” I mean, following Bernard Williams, concepts that have not only a descriptive but also a normative meaning [see 4, p. 129]. Autonomy is such a concept: the description *that* a person, an action, or a decision is autonomous provides practical reasons for one’s behaviour, such as refraining from certain actions towards that person. For further reading see [5].

4 When I speak of rights and duties in the following, I do so primarily against the background of moral considerations and not with a view to the law or the legal standard. However, I am aware of the correlation between moral and legal rights [6, p. 101].

5 I take this term from Jaworska, who cites Alzheimer’s patients and young children as examples of marginal agents [7, p. 529].

6 There are also some national as well as international laws (e.g., Convention of the Rights of Persons with Disabilities, CRPD) that aim to protect the decision-making rights and interests of persons with limited or no capacity. In this context, the Mental Capacity Act (MCA) 2005 of England and Wales is particularly noteworthy. Compared to many other countries where the rights of people lacking capacity are distributed in different laws, including Germany, Canada and France, the MCA brings together the legislation in one detailed document. However, even if the MCA can be considered a milestone in mental capacity legislation, the reality does not always correspond to the law and there is a need for revision after almost 30 years since the beginning of the work on the MCA, as Peter Bartlett [8], among others, points out (see also [9, 10]). This includes, for instance, the outdated understanding of disability, a narrowly rationalist framework of capacity, the omission of mentioning relational autonomy, as well as ambiguities regarding the best interests standard (BIS). In addition, the MCA only applies to persons aged 16 and over. It is therefore only partially relevant to the group of marginal agents I am looking at – namely patients with dementia, but not young children.

7 Of course, it is difficult to make general statements here, as marginal agents form a heterogeneous group that includes patients with dementia as well as small children or patients with intellectual disabilities. The state of research is very different with regard to different patient groups and also countries. Nevertheless, there are empirical studies that point in this direction [22]. Since marginal agents are in general a very vulnerable group of patients, I consider that any hint of disadvantage should be taken seriously and no attempt to strengthen their interests and participation should be omitted.

8 In the following, I will use “duties of autonomy”, “autonomy duties”, “obligations of autonomy” and “autonomy obligations” interchangeably.

9 In simplified terms, autonomous action, according to Kant, is action that is in accordance with the moral law [26, pp. 433, 440–441].

10 In the philosophical debate on autonomy, a distinction is often made between a local and a global perspective on autonomy (see for example [27, pp. 17–19; 28, p. 16; 29, p. 100]). While local autonomy usually refers to the autonomy of a concrete action or decision, global autonomy refers to a more comprehensive understanding of autonomy. It usually describes the autonomy of a person’s way of life. The question then is not whether an individual action or decision is autonomous, but whether a person can shape her life in the light of her own values and according to her own principles. I regard both forms of autonomy as relevant in the context of medical ethics. I also believe that the two forms cannot always be strictly separated and can be mutually dependent. Individual decisions can have meaning in their own right, or they can contribute to shaping one’s life and thus to global autonomy.

11 I am not committing myself to any specific theory here. For a good overview of different understandings and theories of autonomy, I recommend [30] and [31].

12 As previously stated, I will focus on the normative meaning of autonomy as a value – not as a right. Nevertheless, I would like to mention that it also depends on the chosen theory of rights whether and which kind of autonomy rights are granted to marginal agents. According to some versions of the “will theory of rights” that understand rights as the protection of autonomous (in the sense of conscious and deliberate) choices, it is not possible or at least difficult to grant autonomy rights to marginal agents [6, 38].

13 When I speak of “young children” or “children” in the following I mean, in agreement with Amy Mullin, the age group between three and eight years [39, pp. 536–537].

14 Jaworska points out that this has already been proven with regard to children by studies in developmental psychology and even if it has not yet been systematically documented for Alzheimer’s patients, there is a lot of evidence from individual cases [7, pp. 530–531, 565–566].

15 The example serves primarily to illustrate that even young children form these complex emotional attitudes. That they do so is of course also conceivable in the context of therapies and treatment decisions which I will address later in the paper. However, I have deliberately chosen a non-medical example in order to leave out complex issues that result from stronger duties of beneficence towards children and that undoubtedly need to be discussed in their own right.

16 In an earlier version of this paper, I described carings as an important ‘pre-form’ of autonomy. If one assumes that autonomy is not necessarily exercised only through rational or reflexive capacities, but also through affective ones, the term pre-form is no longer appropriate. Carings are rather a particular form or manifestation of autonomy.

17 Kalle Grill argues that what matters is not how competent or voluntary a desire or choice is, but how important it is to the person – a criterion that can also be applied to children (see [44]).

18 There are different understandings of what the BIS encompasses [45, 46]. I draw on a widely shared understanding of the BIS as an overall determination of a patient’s best interests based on beneficence and nonmaleficence considerations, considering both objective well-being goods and subject-relative goods [8, 45–48]. In proxy decision-making, the BIS is used mainly when the patient has never been competent and/or nothing is known about the patient’s preferences [49, 50, 25, p. 142]. Sometimes the BIS is also understood in a more subjective manner in the sense of what a patient would want for themselves if capable (also referred to as ‘substituted judgment standard’ [25, pp. 139–140]) and should reflect their personal values and preferences [8, pp. 4–5; 51, p. 36].

19 “Overall well-being” refers to a comprehensive perspective on well-being that takes a holistic view of what is good for a person, i.e., considers all areas of life. This is to be distinguished from a purely health-related perspective on well-being.

20 The terms “well-being” and “the good life” are often used interchangeably. In my dissertation (forthcoming), I discuss the differences between these two and related concepts. In the following, however, I endorse the synonymous use, as the differences are not relevant for the considerations in this paper.

21 The thought experiment recalls earlier reflections by Jonathan Glover on the desire to take one’s own decisions, especially regarding central aspects of one’s life [58, pp. 80–82]. Another philosophical thought experiment to be mentioned in this context is Robert Nozick’s “experience machine” experiment (see [59, pp. 42–43]).

22 This value is often referred to as the “intrinsic value” of autonomy (e.g., [31, 52, 56, 60–62]). However, Christine Korsgaard rightly points out that this term is misleading if one wants to express that autonomy *is valued as such*. In this context, it is more appropriate to speak of the “final value” of autonomy [63]. For “intrinsic” refers to the source or ‘location’ of the value and thus represents the opposite of “extrinsic” whereas the distinction between “final” and “instrumental value” is a distinction about “two different ways in which something can be desired (or valued)”, as an end or as a means [64, pp. 388–390]. In other words: While the intrinsic/extrinsic distinction is about value as property, the final/instrumental distinction is about value as desirability. In the following, I will therefore speak of the “final value” of autonomy when I refer to the appreciation for its own sake – independent of its contribution to other values.

23 In short, the IC doctrine states that patients have the right to make an informed and personal decision about their own treatment [65]. This requires that patients receive and understand all information before making a treatment decision.

24 My own translation.

25 Most theories of autonomy, including internalist theories, recognise the *causal* importance of social relations for one’s autonomy, i.e., the extent to which one’s exercise and development of autonomy is promoted or hindered by one’s social environment. Relational theories of autonomy, on the other hand, which consider social relationships to be *constitutive* for autonomy assume that certain social relationships are decisive for the autonomy of an action, decision, or person. In a sense, they ascribe to them a definitional role for autonomy [68].

26 To convey information to children participating in studies in a more child-friendly way, Gert Helgesson suggests drawings, photographs, and video recordings, for example. Special forms of presentation can also increase the child’s attention [71, p. 312].

27 For further information, see https://www.hug.ch/accueillir-patient-avec-handicap (accessed 26 January 2023).

28 For a detailed discussion see for example [55, 73, 74]. For the distinct characteristics of children as patients see [75] and as participants in longitudinal studies see [71] (concerns the topic of IC).

29 Betzler understands a “project”, or more precisely a “personal project”, to be an undertaking which “is governed by norms that determinately prescribe the constitutive aim of that project” [55, p. 76]. A “proto-project”, on the other hand, is more like a “project in the making”: connected activities “that, in principle, could amount to a full-blown personal project” [55, p. 79].

30 Beauchamp and Childress make an exception referring to the much-discussed case of the Alzheimer’s patient Margo, whose signs of happiness seem so great that it is justified according to the two authors to override her previously established treatment wishes in favour of her current experiential interests [25, p. 142]. However, they do not explain why an exception is justified here. Regarding the case of Margo, see [2, 81–83].

31 Even though I am explicitly concerned with autonomy rights here, I would like to mention that the attribution of rights is not always linked to certain conditions. This is of course especially valid for human rights and fundamental rights.

32 How extensive this task is, of course, depends on how broadly one defines the concept of health – a discussion I cannot have in the context of this paper (for further reading I recommend [87] and [88]). But even if hardly anyone today would claim that it is a matter of a purely biological-functional understanding of health, for example in Christopher Boorse’s sense [89], this does not change the fact that the goals of medicine continue to be primarily directed towards the elimination of symptoms and the restoration of functional abilities.

33 The various challenges of extending autonomy-enabling duties in patient care would also need to be considered in more detail. One challenge that arises if one wants to take greater account of the carings of marginal agents would certainly be the assessment of authenticity. In order to be able to judge whether a patient’s personal interests and wishes are authentic and important to them, one has to accompany the patient over a longer period of time – which of course is not the case in many physician-patient relationships, rather in caregiver-patient relationships.
